# Experimental peptides, GLP‐1s and polypharmacy: Future directions for image‐ and performance‐enhancing drug harm reduction

**DOI:** 10.1111/add.70545

**Published:** 2026-07-09

**Authors:** Luke A. Turnock, Lambros Lazuras

**Affiliations:** ^1^ School of Social and Political Sciences University of Lincoln Lincoln UK; ^2^ School of Psychology, Sport Science and Wellbeing University of Lincoln Lincoln UK

**Keywords:** GLP‐1, harm reduction, IPED, peptide, polypharmacy, public health

*The rapid growth of experimental peptide and off‐label GLP‐1 use highlights the need for harm reduction approaches that extend beyond anabolic steroid use. Emerging patterns of polypharmacy, unregulated online markets and increasingly diverse user populations require adaptive public health responses, including targeted outreach and education*.

We welcome the suggestion by Piatkowski and colleagues [1] that public health must adapt to the needs of people who use anabolic‐androgenic steroids (AAS), acknowledging the fundamental differences in pharmacology and use patterns that AAS have compared to conventional narcotics. Building on this, we believe the recent explosion in off‐label GLP‐1 medicine and experimental peptide use highlights further the need for public health to adapt to emerging forms of drug use in increasingly diverse populations.

Piatkowski and colleagues [1] note that AAS use has expanded beyond elite sports to encompass a larger, more diverse population. Image‐ and performance‐enhancing drug (IPED) use is increasing, with a growing population of anti‐ageing and wellbeing‐oriented consumers, along with recreational exercisers interested in fat loss or aesthetic enhancement, consuming an expanding range of compounds [2, 3].

Experimental peptides like BPC‐157, TB500 and CJC‐1295 are increasingly available and consumed by a heterogeneous population engaging in recreational exercise that do not always align neatly with prior typologies of AAS use [2, 4, 5]. Peptides may come with longer‐term organ‐based harms that are not currently understood, given the paucity of large human clinical studies on many of these substances [6], and are subject to the same issues with quality and purity that Piatkowski and colleagues [1] identify in relation to AAS. Research suggests illicit market peptides and GLP‐1s are often substandard quality [7, 8], signifying the need for urgent harm reduction interventions like testing services, education and outreach.

Although evidence regarding the effects GLP‐1 receptor agonists in clinical populations is growing, off‐label use may diverge substantially from approved indications and prescribing guidance [9, 10]. Of particular concern is increasing interest in Retatrutide, an investigational agent that has not yet received regulatory approval [11, 12, 13]. Use of unapproved products obtained outside regulated healthcare settings may expose individuals to additional risks related to product quality, dosing accuracy, safety monitoring and the absence of established regulatory oversight. There is also evidence that some individuals purchasing GLP‐1 products online may be unaware that products obtained from unregulated sources may not meet pharmaceutical manufacturing standards or may differ from approved medicines [3].

Piatkowski and colleagues [1] highlight polypharmacy as a high risk behaviour and we have also seen trends in risky polysubstance use among experimental peptide and GLP‐1 users. Our prior research documented the ‘stacking’ of multiple GLP‐1s and their ‘cycling’ in rapid succession, greatly increasing risks of severe negative health events [9]. Although this work related to bodybuilders, our ongoing research suggests non‐subcultural GLP‐1 consumers often engage in similar ‘stacking’ practices to achieve ‘optimal’ weight‐loss or wellbeing outcomes, often with little seeming understanding of associated health risks.

A particularly concerning trend is the prevalence of risky polypharmacy among users of experimental peptides. Although combining BPC‐157 and TB500 was historically recommended by trainers for short‐term use to accelerate injury repair [2], increasingly we are seeing products used that contain three or four different peptides in a single vial or injector pen and individuals using these ‘stacks’ for prolonged periods. Significantly, some peptide users appear to be stacking these peptide ‘blends’ with GLP‐1s, engaging in polypharmacy with five or more compounds that have unknown interactions, magnifying risks substantially.

Combined with further polypharmacy with AAS, selective androgen receptor modulators (SARMs), stimulants or other supplements prevalent in fitness cultures [14, 15, 16], this polysubstance use is likely to be where most harm occurs within IPED using populations. Harm reduction must, therefore, think beyond single categories of drugs and be responsive to broader polypharmacy trends and risks.

Piatkowski and colleagues [1] rightly argue that harm reduction approaches developed around conventional narcotics may not adequately address the realities of AAS use. The rapid growth of experimental peptide and off‐label GLP‐1 use further reinforces this point. These substances are attracting increasingly diverse populations, are often sourced through unregulated markets and used by non‐clinical populations in ways that diverge substantially from clinical guidance. Simultaneously, emerging patterns of polypharmacy create new and poorly understood risks that existing harm reduction frameworks do not effectively address.

As the landscape of IPED use continues to evolve, public health responses must evolve alongside it. Harm reduction should move beyond substance‐specific approaches and recognise the interconnected nature of contemporary IPED use, including the growing importance of experimental peptides, GLP‐1 drugs, digital marketplaces and online communities of users. Equally important is the development of new forms of outreach capable of engaging populations who may never encounter traditional drug services. This is particularly significant in relation to populations accessing pre‐made injector pens or those using oral compounds [3, 17], who may not access needles and syringe programmes, and whose only source of information may be social media.

## AUTHOR CONTRIBUTIONS

**Luke Turnock:** Writing—original draft; writing—review and editing. **Lambros Lazuras:** Writing—original draft; writing—review and editing.

## DECLARATION OF INTERESTS

None.
